# Obituary

**Published:** 1911-12-30

**Authors:** 


### Obituary.

DEATH OF AN EMINENT LEEDS PHYSICIAN.

Dr. Edmond Fauriel Trevelyan, M.D., B.Sc., F.R.C.P., J.P., died recently after an illness extending over several months, at the age of fifty-two. He was taken ill at Aldershot in August, and moved to his home in Leeds, but has been unable to continue his practice. An operation was performed upon him five weeks since, and another shortly afterwards, both of which were successful. A relapse, however, occurred, which resulted fatally.

Dr. Trevelyan was born in Warwick, but spent practically the whole of his professional life in Leeds. He was for some time at St. Bartholomew's, London, and subsequently studied in Vienna and Berlin. In 1894 he was elected Honorary Physician to the Leeds General Infirmary, and continued to hold that position to the last. He was also on the staff of the dispensary, and was connected with all the medical charities of the town. The Leeds Association for the Prevention of Tuberculosis was largely due to his initiation. In 1899 he prevailed upon the then Lord Mayor, Colonel Harding, to convene a conference to consider means for combating tuberculosis, and the result was the establishment of the two Consumptive Hospitals at Gateforth and Armley. Last year he saw the realisation of a scheme of his own in the erection of a Children's Home and School at Gateforth. Dr. Trevelyan succeeded Dr. Jacob in 1894 as Professor of Pathology in connection with the Leeds Medical School, and subsequently Professor of Therapeutics at the University of Leeds. Dr. Trevelyan's reputation extended beyond the confines of the West Riding, for his knowledge of tubercular disease and diseases of the throat and nervous ailments. His literary and scientific gifts enabled him to write extensively and do a considerable amount of revising for the medical journals.

Dr. William Henry Watts has died after a somewhat lengthy illness. He qualified in 1857 as member of the Royal College of Surgeons, having been a student at St. George's Hospital. Dr. Watts was at one time resident medical officer of the Lambeth Lying-in Hospital.

Dr. Francis Thomas Bond has died, after a short illness, at his residence in Gloucester, in his seventy-eighth year. During the last thirty-eight years Dr. Bond had been Medical Officer of Health of the Gloucestershire Combined Sanitary District, and had worked with unceasing energy in the direction of sanitary reform. One of the keenest opponents of the anti-vaccinators, he was frequently to be found championing the cause of compulsory vaccination. He was the honorary secretary of the Jenner Society, in the founding of which he took a prominent part. Dr. Bond was continually engaged in research work, and turned his attention to many other interests with great success. He introduced improvements in a process of cheese manufacture and promoted the study of scientific agriculture, besides inventing a patent hygienic quilt. Dr. Bond pursued his medical studies at St. Mary's Hospital and at Birmingham, qualifying in 1856. He became M.D. Lond. in 1860. For a time he was physician and professor of clinical medicine at Queen's Hospital, Birmingham, and was a Fellow of the Royal Society of Edinburgh. He was a former president of the Midland Association of Medical Officers of Health and of the local branch of the Society of Medical Officers of Health.

